# Influence of liver cancer on lipid and lipoprotein metabolism

**DOI:** 10.1186/1476-511X-5-4

**Published:** 2006-03-03

**Authors:** Jingting Jiang, Peter Nilsson-Ehle, Ning Xu

**Affiliations:** 1 Section of Clinical Chemistry & Pharmacology, Institute of Laboratory Medicine. Lund University, S-221 85 Lund, Sweden; 2 Department of Tumor Biological Treatment, The Third Affiliated Hospital, Su Zhou University, Changzhou 213003, China

## Abstract

Liver plays a key role in the metabolism of plasma apolipoproteins, endogenous lipids and lipoproteins. Hepatocellular carcinoma (HCC) is one of the most common fatal malignant tumors in China and in other Southeast Asian countries. This has been attributed to the high incidence of hepatitis B infection. Hepatitis B proteins, such as the hepatitis B X protein (HBx) that is large hepatitis B surface protein could regulate transcription of many candidate genes for liver carcinogenesis. It has known that patients who suffered from acute hepatitis B could have lipid disorders such as decreased plasma level of high-density lipoproteins (HDL). Furthermore, aberrations of lipid metabolism are often seen in the chronic hepatitis B infection.

Plasma lipid profiles could be changed under HCC. In majority of the reports in HCC, plasma levels of triglycerides (TG), cholesterol, free fatty acids (FFA), HDL, low-density lipoproteins (LDL), lipoprotein (a) (Lp(a)), apolipoprotein AI (apoAI) and apoB were slight to significantly decreased, however, in some cases plasma levels of TG and Lp(a) might be increased. It has been suggested that analysis of plasma levels of lipids, lipoproteins and apolipoproteins in the patients suffered from HCC reflects on the hepatic cellular impairment status. Studies revealed that alterations seen in the plasma levels of lipids, lipoproteins and apolipoproteins reflecting patients' pathologic conditions. Decreased serum levels of cholesterol and apoAI may indicate a poor prognosis.

Human leukaemic cells and certain tumor tissues have a higher receptor-mediated uptake of HDL and LDL than the corresponding normal cells or tissues. LDL and HDL have therefore been proposed as a carrier for the water-insoluble anti-cancer agents.

## Introduction

Liver is one of the most important organs in energy metabolism. Most plasma apolipoproteins, endogenous lipids and lipoproteins are synthesized in the liver [1,2], which depends on the integrity of cellular functions of liver [2,3]. Under normal physiological conditions, liver ensures homeostasis of lipid and lipoprotein metabolism [4]. Hepatic cellular damage and HCC impairs these processes, leading to alterations in plasma lipid and lipoprotein patterns. Mortality due to liver cancer is the fifth common malignant tumor worldwide [5], and it is closely related to the infections of hepatitis B virus (HBV) [6-8] and hepatitis C virus (HCV) [9]. HBV proteins, such as the hepatitis B X protein (HBx) that is large hepatitis B surface protein could regulate transcription of many candidate genes for liver carcinogenesis [10,11]. As HBV and HCV infections are quite common in China and in other Southeast Asian countries [12-14], the mortality of HCC is 20,4/100,000 in the population of China, corresponding to about 18.8% of all fatal malignant tumors [12]. Hepatic diseases differ from most other causes of secondary dyslipidemia in that the circulating lipoproteins are not only present in abnormal amounts but they frequently also have abnormal composition, electrophoretic mobility and appearance [15]. It has been demonstrated that patients suffered from acute hepatitis B could have lipid disorders, for instance, decreased plasma HDL [16,17]. The aberrations of lipid metabolism are often seen in the chronic hepatitis B infection too [18]. HDL and its major apolipoproteins, apoAI and apoAII, are frequently reduced in the patients suffered from cirrhosis or HCC [15]. Decrease in the level of serum LDL cholesterol in patients with liver disease was significantly correlated to the increasing severity of the disease [19-22]. In the present review we discussed aberrations of lipid profiles in the patients suffered from HCC. Decreased serum levels of cholesterol and apoAI may indicate a poor prognosis [22-24]. The changes of lipid profiles under HCC are listed in table 1.

**Table 1:** Summarization of serum lipid/lipoprotein changes under HCC

**Lipids/Lipoproteins**	**Changes under liver cancer**	**References**
**Triglycerides**	**↑** **↓↓ or-**	[26] [21], [22]
**Total cholesterol**	**↑** **↓ to ↓↓**	[26] [19], [21], [22], [36]
**Free fatty acids**	**↑**	[52]
**Lp(a)**	**↑** **↓ to ↓↓**	[75] [21], [72], [73]
**HDL**	**↓↓**	[19], [21], [22], [36], [79]
**LDL**	**↓**	[19]
**ApoAI**	**↑ **(proapoAI) **↓↓**	[81] [19], [24], [23], [82]
**ApoAII**	**↓↓**	[24], [23]
**ApoB**	**↓**	[83]
**ApoM**	**↓↓ **Hepatic expression	Unpublished data

↑ slight increase; ↑ slight decrease; ↓↓ significant decrease; – no change.

### Influence of HCC on metabolism of triglycerides, cholesterol and free fatty acids

The patients with HCC frequently have other liver diseases such as chronic hepatitis and cirrhosis. All these conditions (hepatitis and cirrhosis of the liver) are often associated with plasma lipid and lipoprotein aberrations [25]. It has been demonstrated that plasma triglycerides (TG) decreased by 20–30% in the patients with HCC [21]. In contrast, Alsabti [26] reported that serum TG in HCC patients were increased when compared to those with cirrhosis. Ooi, et al., [22] reported that plasma TG levels in HCC patients were not significantly different compared with controls. These results emphasize the fact that changes of plasma lipid profile may not always imply the presence of HCC and one need to exercise caution in interpreting these results.

It is known that lipids and lipoprotein metabolism could be regulated by cytokines. For instance, interleukin-6 (IL-6), tumor necrosis factor (TNF-α), IL-1 may inhibit TG synthesis [27]. Tumor cells are known to produce large amounts of pro-inflammatory cytokines that, in turn, may suppress plasma TG levels. Argiles, et al., reported that IL-1 profoundly affects lipid metabolism by delaying intestinal absorption and decreasing tissue uptake [28]. IL-2 could induce severe hypocholesterolemia that is mediated by the inhibition of lecithin:cholesteryl acyltransferase (LCAT) activity [29]. IL-1 and IL-6 significantly decreased microsomal triglyceride transfer protein (MTP) mRNA levels in HepG2 cells [30]. It is believed that MTP is related to the synthesis of very low density lipoprotein (VLDL) [31]. In addition, these cytokines could also decrease lipolysis *in vivo *[32]. Similar results have been reported in other types of cancers [33].

About 80% endogenous cholesterol are synthesized in the hepatocellular microsomes that contain cholesterol synthesis enzymes [34,35]. In HCC and chronic liver diseases the synthesis and metabolism of cholesterol are impaired. It leads to a decrease in plasma cholesterol levels [19,21,22,36]. Li reported [37], in a forward investigation, the relationship between serum cholesterol and occurrence of cancers in 9021 employees aged from 35 to 64 years old. No evidence of association of serum cholesterol level with total cancer mortality was seen by Log-rank trend test. But there was a significant negative correlation between serum cholesterol level and HCC (P < 0.05). This negative correlation also existed between serum cholesterol level and chronic hepatitis and liver cirrhosis. Similar results were described in patients with gastrointestinal cancer, however, lower cholesterol levels are not related to the cancer stages [38]. Decreased serum cholesterol concentrations were also found in other cancers [39-41], which are probably related to the increased consumption of cholesterol by the tumor cells, as cholesterol levels in the hepatoma tissues were doubled compared to the control tissues [42]. In addition, it has been reported that synthesis of cholesterol is reduced under cancers [43], and moderate increases of serum cholesterol levels and increased body mass index (BMI) may have a protective effect on cancer mortality [44,45]. It was observed that the plasma HDL-cholesterol was inversely correlated to the cholesterol levels in the tumor tissues in cancer patients [46]. Because the use and storage of cholesterol are increased within the tumor tissues during growth, it is possible to hypothesize that lower HDL levels observed in patients with gastrointestinal cancer are associated with the increased cholesterol metabolism in these proliferating tissues [46].

Liver is a key organ for the metabolism of free fatty acids (FFA) [47], and FFA are the source of TG synthesis in the liver [48]. The extracellular FFA pool in tumors undergoes continuous turnover, utilizing adipose tissue storage as a source [49]. It is reported that plasma FFA are increased in tumor bearing animals [50]. Increased plasma FFA is attributed to the hypermetabolic state in cancers [51]. Li, et al., [52] reported that proportions of plasma saturated and monounsaturated fatty acids in the HCC patients were significantly increased compared to the controls. Similar results were found in the patients with breast cancer suggesting that this could be a common finding in tumors [53]. It is believed that these FFA could be used as metabolic substrates by the tumor cells [54]. Hanai, et al. [55] evaluated seventeen hepatectomized cases (12 cases of HCC and 5 cases of metastatic liver cancer). In HCC tissues, the levels of alpha-linolenic acid (LA) and docosahexaenoic acid (DHA) were significantly less than those in the reference tissues those normal tissues surrounding tumors. Wood, et al., [56] analyzed total lipid extracts of liver tissue from 14 HCC patients and demonstrated that ratio of saturate C18 to unsaturated C18 in the tumor tissues were significantly and consistently lower than those corresponding non-tumor tissues. Palmitic acid was the most representative saturated FFA, while unsaturated FFA was represent, in decreasing order, by oleic, linoleic and arachidonic acids (AA) [57]. Available evidence [58] is consistent with the possibility that selective changes in the hepatocellular metabolism of long-chain fatty acids may contribute significantly to the activity of the extramitochondrial pathways, which may also contribute to liver injury and tumorigenesis. De Alaniz and Marra [59] demonstrated a significant contribution of the stearoyl-CoA desaturase system to the high levels of oleic acid (OA) present in hepatoma cells. Peroxisome proliferators are diverse group of chemicals which are regarded as rodent hepatocarcinogens and/or liver tumor promoters [60]. It has been demonstrated that peroxisome proliferators could increase hepatocyte proliferation and cause liver tumors in rodents, which is related to transcriptional activation of peroxisome proliferators-activated receptor (PPAR)-α regulated genes and the resulting excessive generation of H_2_O_2_. Evidence from mice lacking fatty acyl-CoA oxidase (AOX), PPAR-α and PPAR-α/AOX has confirmed the role of PPAR-α in the development of HCC [61-63]. As PPAR-γ functions as a regulator of cell survival and growth in the HCC, PPAR-γ therefore represents a putative molecular target for chemopreventive therapy or inhibition of HCC growth [64]. In addition, decreased expression of hepatic PPAR-α functions as one mechanism underlying the pathogenesis of HCV infection, PPAR-α may serve as a new therapeutic target in traditional treatment of HCV-induced liver injury [65].

### Plasma lipoprotein pattern in the patients with HCC

Liver represents the main site of lipoprotein (a) (Lp(a)) synthesis [66-68]. Half-life of Lp(a) is about 3.3–3.9 days in human plasma [69], which is influenced in the early stage when liver function was impaired [66]. Lp(a) is synthesized and metabolized independently of other plasma lipoproteins, and Lp(a) level is not influenced by various dietary manipulations [70]. Motta, et al., [21] demonstrated that Lp(a) levels were significantly lower in the HCC patients (5.7 ± 2.08 mg/dl) than in the controls (16.78 ± 16.24 mg/dl). Higuchi, et al., [71] elucidated that influences of serum Lp(a) levels in some tumors could be characterized by the production and deliverance of cytokines. It has been reported that serum Lp(a) were significantly lower in the HCC patients [72,73]. However, Geiss, et al., [74] observed patients with acute hepatitis showed a marked increase in Lp(a) concentration, i.e., 7 mg/dL in acute stage and 32 mg/dL in the convalescence of the disease. Basili, et al., reported that Lp(a) could also be increased in the patients suffered from HCC together with cirrhosis [75]. It has been demonstrated that Lp(a) together with ferritin and alpha-fetoprotein could be a sensitive and early marker to evaluate liver function [21]. As Lp(a) has positive correlation with the hepatic status, it has been suggested that Lp(a) could be considered as a index of liver function [21,68,76].

The origin and fate of HDL are less well understood than other lipoproteins. HDL may be formed both in the intestine and in the liver. During lipolysis of TG-rich lipoproteins, HDL particles are also formed. ApoAI and apoAII are the major apolipoproteins of the HDL. Production rate of apoA-I is an important determinant of the variability of plasma HDL concentrations. It is influenced by many factors and apoA-I transcriptional regulation has an impact on plasma HDL concentrations. Nutritional interventions such as, a switch from high-carbohydrate to a high-fat diet appears to exert their major effect on the production rate of apoA-I rather than on clearance [20,77]. HDL plays a key role in the reverse cholesterol transport pathway (RCTP) [24,78]. It has been demonstrated that HDL fraction offers a new approach to study liver diseases [19]. Ahaneku, et al., [36] analyzed HDL-fraction levels including HDL-cholesterol (HDL-C), HDL-phospholipids (HDL-PL) and the ratio of HDL-C/HDL-PL, in HCC patients and compared with the controls. They found that plasma HDL-C, HDL-PL and HDL-C/HDL-PL were significantly lower in HCC patients than those in the controls. Motta, et al., [21] studied 40 patients with HCC, and evaluated the LDL-C, HDL-C. In patients with HCC, LDL-C level was significantly lower than in the controls, but HDL-C did not show a statistically significant difference to the controls. Kanel, et al., [79] reported that patients with primary or metastatic liver cancer had strikingly decreased HDL-C. Ooi, et al., [22] suggested that HDL-C may be clinically useful to reflect the pathologic conditions, and can be used to evaluate the severity of liver diseases. In the metastatic liver cancer showed a lower HDL-fraction level too, even lower than those in the HCC patients. Also, Fujii, et al., [23] found existence of HDL with an abnormal apoprotein composition or a more profound decrease of HDL3 than those of HDL2 in severe hepatocellular dysfunction of cholestasis. Cooper, et al., [19] observed that there was a rapid decreases of HDL-cholesterol and LDL-cholesterol immediately after hepatic resection. Lipid profiles are different in the cirrhosis patients with or without HCC. In cirrhosis with HCC plasma phospholipid levels showed a significant negative correlation with total bilirubin and alanine aminotransferase. Total cholesterol (TC), phospholipids (PL) and the ratio of TC/PL were elevated, while HDL-C, HDL-PL, HDL-C/TC and HDL-PL/PL were normal. It is suggested that variations in the level of plasma lipids and lipoproteins may assist in describing the nature of these two forms of liver disease [36].

### Influence of HCC on apolipoproteins

Liver is the main organ for the synthesis, storage, transportation and degradation of some apolipoproteins [20]. Each protein may be influenced by liver disease in a different way, and serum lipoprotein concentrations with faster turn-over are more reduced with respect to those with slower turn-over [80]. Serum concentrations of apoAI and A-II were significantly lower in the patients suffered from HCC [23,24], but an increase in the proportion of proapoAI was found in patients with HCC [81]. The proportion of proapoAI showed a tendency toward increase under advanced liver damage because liver participates in the process of converting proapoAI to the mature apoAI. It is suggested that plasma apoAI could reflect the hepatocellular dysfunction [2,19,82]. The pattern of changes in the serum apoAI levels may be a good indicator of the hepatic protein synthetic ability during the perioperative period after hepatectomy [82]. ApoB in the liver was an important glycoprotein for transportation of VLDL and LDL, in liver cells hyperexpression of HBx caused accommodation of MTG, HBx could increase the expression of beta-d-mannoside-,-N-acetylglucosaminyl transferase-III (GnT-III), and it could inhibit apoB secretion and enhanced the accumulation of intracellular triglyceride and cholesterol [83]. Ooi et al., [22] reported that slow alpha HDL appeared in the metastatic liver cancer in the early-middle stages, during slight bile stagnation, and accompanied by increases of apo E levels [84]. In our preliminary study we have observed that apolipoprotein M (apoM) mRNA levels were significantly lower in the HCC tissues than those in the normal hepatic tissues surrounding tumors (non-published data). Up to date there is no data reported concerning other apolipoproteins in relation to liver cancer.

### Using HDL and LDL as carriers for the water-insoluble anti-cancer drugs

HDL transports cholesterol to liver cells, where they are recognized and taken up via specific receptors. Cholesteryl esters within HDL are selectively uptaken by hepatocytes via the scavenger receptor class B type I (SR-BI). An interesting feature of SR-BI is that the receptor selectively translocates HDL-cholesteryl esters from the lipoprotein particle to the cytosol of the liver parenchymal cells without a parallel uptake of the apolipoproteins and this property may allow for the delivery of its loaded drugs avoiding lysosomal degradation [85]. As HDL and LDL have high affinity and could be accumulated in tumor cells [86-88], they have been used as carriers for delivery of anti-tumor drugs into hepatoma cells to treat HCC [85,89-93]. Masquelier, et al., [94] investigated the possibilities to use LDL as a drug carrier to increase the selectivity of anti-tumor drugs in cancer chemotherapy. It has been demonstrated that both HDL and LDL may be used as endogenous targeting carriers into tumor cells, which have high lipoprotein-receptor activities, in animal models [88,95]. Anti-cancer drug-HDL complexes work as efficient drug delivery vehicles due to the ability of cancer cells to acquire HDL core components [89,96]. Complex of anti-cancer drugs with HDL and LDL does not influence characteristics of the drugs [92]. Lacko, et al., demonstrated that administration of anti-cancer drug-HDL complex may reduce toxic side-effects during the chemotherapy [89]. Lou, et al., reported in a cell culture system that cellular uptake of recombinant HDL-aclacinomycin (ACM) by the SMMC-7721 hepatoma cells was significantly higher than that of free ACM at the concentration range of 0.5–10 μg/mL (P < 0.01). Cytotoxicity of recombinant HDL-ACM to SMMC-7721 cells was significantly higher than that of free ACM at concentration range of less than 5 μg/mL (P < 0.01) and IC50 of recombinant HDL-ACM was lower than IC50 of free ACM (1.68 nmol/L vs 3 nmol/L) [85]. Chu, et al., coupled doxorubicin (DOX) to human LDL to form a LDL-DOX complex. When the complexes injected into mice, LDL-DOX was more accumulated in liver than free DOX. In contrast, LDL-DOX was less accumulated in heart than free DOX [97]. It suggested that both HDL and LDL could be used as carriers to conjugate water-insoluble anti-cancer drugs leads to a higher accumulation of the drugs locally and specifically.

## Conclusion

It is thus evident that liver plays a vital role in the production and catabolism of plasma lipoproteins and apolipoproteins. Plasma lipid profiles could be changed in HCC. It has been summarized in the Table 1. Analysis of serum levels of lipids, lipoproteins and apolipoproteins in the patients suffered from HCC may reflect the condition of hepatic cellular impairment, and may also be used as an indicator to evaluate patient's prognosis. It is suggested that variations in the levels of plasma lipids and lipoproteins may assist in describing the nature of HCC with or without cirrhosis. The serum apoAI and Lp(a) levels may considered as the index of liver impairments under chronic or HCC. In addition, HDL and LDL had been used as a carrier for delivering chemotherapeutic drugs in HCC and other cancers.

## Abbreviations

HDL, high density lipoprotein; VLDL, very low density lipoprotein; LDL, low density lipoprotein; HCC, hepatocellular carcinoma; Lp (a), lipoprotein (a), HBx, X protein of hepatitis B virus; GnT-III, beta-d-mannoside-,-N-acetylglucosaminyl transferase-III; apoB, Apolipoprotein B; Lp-X, lipoprotein X; FFA, free fatty acids; OA, oleic acid ; AA, arachidonic acid; DHA, docosahexaenoic acid; SR-BI, scavenger receptor class B type I.
